# An Essay on the Therapeutic Value of Certain Articles of the Materia Medica of Recent Introduction

**Published:** 1868-04

**Authors:** John H. Griscom

**Affiliations:** New York City


### AN ESSAY ON THE THERAPEUTIC VALUE OF CERTAIN ARTICLES OF THE MATERIA MEDICA OF RECENT INTRODUCTION.

By JOHN H. GRISCOM, M.D., of New York City.

Read before the New York Medical Society, Feb. 4, 1868, and Reported for the *Philadelphia Med. and Surg. Reporter.*

### SULPHITE OF SODA.

In view of the serious responsibility imposed upon all practitioners of medicine, and actuated by the consideration of the duty belonging to each one to keep his colleagues advised of every additional item of experience that may from time to time accrue to him, I solicit the favor of a few minutes attention of this society, for the presentation of the results of an extended and successful experience with the employment of two articles of the materia medica, of comparatively recent introduction. I refer to the *sulphite salts of soda*, and to *glycerine;* the first as an *internal*, and the second as an *external* remedy; with the first named, I have, during the past two years, had a most gratifying successful experience in a large variety of disorders, finding it to afford relief in very numerous cases in which the usual articles of the materia medica, adapted to previous experience, have sometimes failed, but in which this article has failed in scarcely a single one of over three hundred instances of its administration in those cases to which, according to my theory of its modus operandi, it was specially adapted.

The chemical composition of this material is as simple as that of any other salt—its two ingredients, *sulphurous acid* and *soda*, being well known. The acid constituent, in its separate form, has been long appreciated as one of the most effective antiseptics and deodorizers when used externally. In fact, it is one of the most ancient disinfectants, having been originally employed in its gaseous form, generated by the simple combustion of sulphur. As an antiseptic, a preventer of decomposition and of fermentation, and as a sustainer of the natural composition of nearly all vegetable and animal materials, it appears as useful in the interior, as in the exterior of the organization. On this principle, its efficacy as a remedial agent is chiefly founded, but I have been led to the conclusion that it has an additional value as a prompter of digestion, in cases in which the gastric juice may be deficient in some of its acidulous ingredients.

Preliminary to the discussion of the modus operandi of this article, I ask attention to a few remarks upon the fundamental nature and character of diseases in general. Of the numerous branches of medical science, which it is the duty and the interest of its practitioners to investigate and become well acquainted with, having a bearing on treatment, there is none of more profound, complicated, and important character, than the

ETIOLOGY OF DISEASES.

Upon familiarity with this department of professional knowledge, depends in a large degree the capability of addressing remedial, as well as preventable measures. This subject applies not only to the *external* causes of disturbance of the physiological functions, but likewise to internal sources, in other words, to the pathological conditions provocative of the symptoms indicative of deranged action.

Every practitioner of experience is familiar with the existence of a great number of disorders whose remote origin, and true pathological character, are difficult of comprehension, although their general characteristics and tendencies are well understood, as derived from repeated observations.

The mechanical organization and arrangements of the numerous and complicated structures which compose the animal body, and the physical operations by which the functions of the various organs are carried on, are as well understood by the anatomist and physiologist, as are those of the steam engine, or any other apparatus, by the mechanic and engineer,—and as a general rule, the mode of treatment of almost any particular disease, is as plain to an experienced practitioner as is the management of a sailing vessel in a storm to an experienced seaman. There are occasions when the force of the winds, the blasts of lightning, and the turmoil of the sea, are impossible to be overcome, especially with a vessel of frail structure, whereby its loss becomes inevitable. So with the human constitution; its organic structure is frequently so feeble, and the opposition to the elements of life are so utterly overwhelming, that neither nature nor art is able to control them, and in a considerable number of the derangements with which humanity is afflicted, the *remote* causes are as little known and understood by the physiologist, as are the causes of hurricanes, and of thunder by the navigator.

Some diseases are known to be of a strictly specific character, derived from the introduction into the organism of some peculiar substance to which the idea of *poison* is veraciously applied, and whereby the natural functions are disturbed, and in many instances so extensively deranged, as seriously to impair the vital powers, and for the modification or counteraction of which, what is known as *specific treatment* is required. As examples of this class, we may refer to syphilis, variola, intermittent, and yellow fever.

The *chemistry of physiology* must be regarded as the true quarter whence the real nature of most idiopathic diseases is to be learned. There exists no other structure so complicated, delicate, and numerously varied in its chemical combinations. In none other is there to be found so large a number of the primary elements of nature; in no other laboratory is there so great a number and variety of operations; and it is owing either to the presence of some foreign matter, to the deficiency or surplusage of one or more of the natural ingredients, or to their mal-combination, that a majority of the diseases are to be properly attributed.

Carpenter, in his work on the Principles of Human Physiology, page 535, very properly remarks:

"From the part which the blood performs in the ordinary process of nutrition, it cannot be doubted that it undergoes important alterations when these processes take place in an abnormal manner. The alterations must be sometimes the causes, and sometimes the effects, of the morbid phenomena, which constitute what we term diseases. Thus when some local cause affecting the solid tissues of a certain part of the body, produce inflammation in them, their normal relations to the blood are altered; the consequence is that the blood in passing through them, undergoes a different set of changes from those for which it is originally adapted, and thus its own character undergoes an alteration, which soon becomes evident throughout the whole mass of the circulating fluid, and is, in its turn, the cause of morbid phenomena in remote parts of the system. On the other hand, the strong analogy between many constitutional disorders, and the effects of poisonous agents introduced into the blood, appears clearly to point to the inference that these diseases are due to the action of some morbific matter which has been directly introduced into the current of the circulating fluid, and which has affected both its physical and its vital properties."

The symmetry of many diseases which do not immediately depend upon external causes, necessarily involves the idea of the presence of a morbific agent in the circulating fluid. For example, palsy from lead—the agent known to be mingled with the blood, and to be deposited in the parts affected. Among the most frequent causes of depravation of the blood is the retention in it of matters which should be removed by the excretory processes; *e. g.*, carbonic acid, if totally retained by the lungs, will occasion death in a few minutes. The retention of the materials of the biliary and urinary secretions is a fertile source of disorders of the system—often of fatal results.

The most remarkable cases of depravation of the blood by the introduction of matters from without, are those in which these matters act as *ferments*, exciting such chemical changes in the constitution of the fluid, that its whole character is speedily altered, and its vital properties impaired. Of the causes of blood depravity, one of the most prolific, direct, and most continually in operation, is that function upon which the entire structure of the body is dependent for its renewal and continuance, *viz.*, digestion and assimilation, in which must be included respiration, this being, in fact, the last act of digestion. Very numerous diseases are well known to derive their existence from either the imperfection of this function, or from the inclusion of improper material.

In Draper's Physiology, we are informed, page 77:

"The imputed control which the alkalinity or acidity of the digestive juices exerts in determining the result, illustrates the important function discharged by common salt, which furnishes to the juices of the stomach and intestines the characteristic ingredients they require, by breaking up readily into hydrochloric acid and soda, and *re*-forming at once whenever these materials are brought in contact."

Carpenter, in the work before quoted, remarks, page 503:

"This fluid (gastric juice) of which the existence has been denied by some physiologists, is not very unlike saliva in its appearance; it is, however, distinctly acid to the taste; and chemical analysis shows that it contains a considerable proportion of free muriatic acid, and also some acetic acid. The former must evidently be derived from the decomposition of the muriate of soda contained in the blood, the remote source of which is the salt ingested with the food. Besides these principle ingredients, the gastric fluid contains muriates and phosphates of potash, soda, magnesia, and lime."

Prof. Dunglison, in his work on Human Physiology, states:

"*The quantity of free hydrochloric acid was surprising;* on distilling the gastric fluid, the acids passed over, the salts and animal matter remaining in the retort; the amount of chloride of silver thrown down, on the addition of nitrate of silver to the distilled fluid, was astonishing."

Thos. Chambers, M.D., in his work on Digestion and its Derangements, remarks:

"It may be considered settled that for *healthy digestion, an excess of acid is necessary.* In dogs it may be considered as settled by the experiments of Drs. Bidder and Schmidt, that the necessary free acid in the healthy state is the hydrochloric. This is the result of eighteen experiments made upon the gastric juice of the animals named, after fasting from eighteen to twenty hours, in all of which there was found free hydrochloric, and not a trace of any organic acid. On the whole, the evidence at present seems to point to the conclusion, 1st, that most probably free *hydrochloric* acid is normally present in the healthy gastric juice when it freely exudes on the presence of a natural stimulus; 2d, that it is present in smaller quantities in man than in dogs; 3d, that in man it may be easily neutralized and rendered undiscoverable by the gastric juice being overpowered by a comparative excess of mucus or saliva, normally in excess in fasting, abnormally so in disease."

M. Schmidt, by recent observations made on the gastric juice of a woman having a gastric fistula, calculated that 580 grammes (about 24 ounces) were secreted in an hour, which would give 14 killogrammes (about 36 pounds) a day, being about one-fourth the weight of the body. The following is given by M. Schmidt as the result of nine analyses of the gastric juice of the dog, obtained as pure as possible; 1000 parts contained:

| | |
|---|---|
| Water, | 973.062 |
| Organic matter, | 17.127 |
| Phosphate of lime, | 1.729 |
| " magnesia, | .226 |
| " iron, | .082 |
| Free hydrochloric acid, | 3.030 |
| Chloride of potassium, | 1.125 |
| " sodium, | 2.507 |
| " calcium, | .624 |
| Chloro-hydrate of ammonia, | .468 |

According to M. Velpeau, it is permanently acid in different degrees, a fact established by the experiments of Leuret, Lassaigne, Tiedeman and Gmelin.

Considering the exceedingly complicated character of this fluid, and the very profound nature of the chemico-physiological operations it is required to undergo in combination with the multiplicity of material imbibed as food and condiment, and it being the primary foundation of the "house we live in," it is clear that any deficiency in its chemical ingredients, or the presence of any material in the stomach upon which it cannot properly act, must result in deficient nutrition of the blood, and consequently of various parts, and possibly of the whole structure of the body.

To whatever circumstance indigestion may be owing, whether to deficiency of amount of gastric juice, the privation of some of its essential ingredients, or to a defective capacity of the circulatory or nervous functions, (upon the integrity of which this function is also largely dependent,) the contents of the stomach must undergo the decomposition and fermentation, which invariably occur with all dead animal and vegetable matter when confined in vessels where they are subject to the continued influences of heat and moisture. To this natural operation are due the generation of gases which give the symptom of flatulence, pervading both the gastric and intestinal organs; also the nausea and eructation of the food, so frequent in dyspepsia; and likewise the epigastric pains, so distressing in many instances; together with the diarrhœa, dysentery, colic, and hepatic congestion and torpor, so frequently resulting from the presence of the foreign masses thus retained, and imparting, to a greater or less extent, foul and injurious ingredients to the blood by absorption.

My experience with the remedy referred to in the treatment of diarrhœa, dysentery, cholera-morbus, as well as dyspepsia, has been most decidedly beneficial. From five to twenty, forty, or sixty grains, according to the age of the patient and the severity of the symptoms, administered two, four, or six times a day, have, in almost every instance, had the effect of speedily arresting the discharges, and relieving the nausea and the colicky irritation. I could cite several cases in which its efficacy has proven as prompt as any other remedy before tried, and in not one have I seen any bad effect or failure. As to its *modus operandi* in these complaints, it seems to act in the double capacity of an antiseptic and astringent. On the latter principle, its influence appears sometimes almost as speedy and efficacious as opium. In cases of constipation derived from torpor of the liver, or deficient peristalic power of the intestinal tube, its corrective influence over almost all functions aids to restore a healthy action of the muscles of the bowels.

In dyspepsia its efficacy has been most marked, especially when the disorder is accompanied with flatulence and eructations of food. These symptoms are doubtless the result of the decomposition and fermentation of the foreign material in the stomach itself, from one or more of the causes before mentioned. In such cases the sulphite salt operates, in the first place, as a direct and powerful arrester and preventive of the decomposition of the food, in the same manner as it does on the outside of the body; and, in the second place, its acid consituent, either in its original sulphurous form, or by its advancement to the sulphuric form, doubtless compensates for some of the deficiency of the gastric juice, and in this way completes the digestive process as far as the gastric function is concerned. The form of administration which I have found most useful and successful in dyspepsia and its attendant circumstances, is in combination with tonics and carminatives, avoiding alcoholic stimulants on all occasions. My chief combination is tincts. of cinchonæ comp., and cardamoms, and syr. aurantii, with the sulphite salt in separate solution, combining the two at the time of administration.

It speedily arrests the fermenting process which the contents of the alimentary canal so frequently undergo, eliminating gases, producing symptoms of flatulence, and which doubtless in many cases is the cause of the diarrhœa, nausea, colic, and other attendant symptoms. For the arrestation of this process of decomposition and fermentation, I have found no means equal to sulphurous acid in the form of a sulphite salt. In several instances in which flatulence was a very prominent symptom, one or two doses of the salt appear to have immediately arrested and removed it. As an illustration of its value in dyspepsia, the following extract is quoted from a letter received from a recent member of the General Government in Washington:

"*Washington City, Jan. 8, 1867.*

"DR. J. H. GRISCOM.

"*Dear Sir*—You remember your medical prescription. I procured it; and from taking the first dose, I felt no more of that dyspeptic trouble. I took faithfully the twelve powders and the liquid, and believe they have been of more benefit to me than all the medicine I have taken for years. Since then, several of my friends have been complaining, in my presence, of the same trouble I had, and I have immediately given them the apothecary's number of your prescription, and in the only report I have had, it cured the gentleman just as it did me. I am not sure I shall not set up 'Doctor for Heartburn,' on your capital."

There is another disorder in which the intestinal canal is chiefly involved, and accompanied with other serious disturbances, in which, though it has not fallen to my lot during the past two years, to have obtained any experience in the treatment with this or any other remedy, having seen no case of it, I yet should unhesitatingly and with full confidence, administer it. I refer to *cholera Asiatica.*

Sulphuric acid, diluted in the form of a beverage, has gained, in France, considerable reputation in the treatment of epidemic cholera, its remedial effects being attributable to its antiseptic powers, and to its influence in destroying cryptogamic parasites and organic germs. In Sulphur*ous* acid we have a preparation of the same ingredients in different proportions, possessing the same advantages combined with the additional one of being a powerful antiseptic and deodorizer, in its native form, and then, by conversion into sulphur*ic* acid, possessing all the advantageous properties of the latter.

In any disease whatever, in which the digestive function is impaired, whereby the recuperative faculties of the entire system must be more or less diminished, the capacity of the sulphite in arresting the decomposition and fermentation, and thus preventing the additional trouble and increase of derangement necessarily resulting therefrom, is a most valuable *adjuvant.*

A marked illustration of this theory is found in typhus fever and other analogous disorders. In that species of derangements, the digestive powers are manifestly reduced, at the same time they are our principal reliance for the addition of the antitoxic remedies to the circulating fluid.

In the diseased condition known as *Scorbutus*, there is a most direct demand for proper alimentary material, and therein we find the sulphites valuable, not only as a means of suspending the fermenting process, but also, by the agency of both its acid and alkaline constituents promoting digestion itself.

But it is not alone upon the contents of the stomach and bowels, with which the salt comes in direct and immediate intercourse, that its antiseptic and antizymotic influence is exerted. This, as before suggested, is probably due to the action of the sulphurous acid derived from the decomposition of the salt. But there are many diseases of a zymotic character, derived from causes wholly independent of the digestive function, upon which this agent has been found to exert a curative influence as rapid and efficacious as in those already referred to.

We have several reports in medical journals, of its efficacy in intermittent and typhus fevers, in scarlatina, small-pox, and measles, the theory of its action in which is, that the acid is absorbed into the blood itself, and therein exerts its antiseptic properties directly upon the materies morbi which give rise to the disorders.

Even in yellow fever, the real chemico-physiological cause of which has not yet been satisfactorily made known, it was last summer, during the prevalence of that disease in the West Indies, reported by the medical officers of the British fleet, to have produced highly favorable results.

Its value in erysipelas I have had the gratification of testing in several cases. In one case in the New York Hospital, found on the face of a delirium tremens patient, a few doses of the salt wholly relieved that symptom in twenty-four hours. In this peculiar disorder there would seem to be a very plain reason for its usefulness, it being a disease whose source is most plainly derived from internal derangement of the blood, producing obstruction of the functions of the capillary circulation of the skin, thus giving rise to congestion and inflammation. That it is derived from some chemico-pathologic alteration of the blood, there can be no doubt, although we know not the true nature of the change.

In several cases of bronchial and pharyngeal catarrh, I have also observed singularly beneficial results from its administration in connection with local treatment, this disorder being considered as based upon the same foundation as cutaneous ery sipelas.

The origin of many cases of idiopathic pneumonia, peritonitis, pleuritis, and other disorders embraced in the same nosological class, is a question of the highest scientific importance, both in relation to the nature of the disease and its treatment. In the remarks of Carpenter, before quoted, relating to the changes produced upon the blood by *local causes*, thus giving rise to morbid phenomena in distant parts of the system, we have an intimation of the influence of the alterations of the blood disturbing the capillary functions; whence it is easily perceivable that the so-called inflammatory affections just named may be the direct effects of morbific changes in the blood, introduced by other than local causes. In the treatment of these diseases and others of similar character, such as erysipelas, gastritis, enteritis, hepatitis, and all others of idiopathic origin, it is rational to look into the chemical condition of the vital fluid, both for their origin and for the means of restoring it to its healthful condition. There is one very common disorder, the chemical origin and cause of which is now almost universally admitted, and for which chemical anti-toxic treatment is very generally found available. I refer to rheumatism, which is known to be dependent upon a hyper-acetic condition of the blood, and capable of neutralization by the use of alkaline medicines. The alkaline salt, known as tart. pot. et sod. has proved in latter years to be an almost uniform remedy for all forms of this disease. In a recent paper on this topic by Professor J. H. Salisbury, of the Charity Hospital Medical College, Cleveland, Ohio, contained in the last October No. of the *Amer. Jour. of Med. Sciences*, we are presented with a very lucid exposition of the several varieties of this disorder illustrated by microscopic expositions. He remarks: "We have four or more types of rheumatism, which may be designated as follows: 1st, Lithic; 2d. Oxalic; 3d. Cystinic; 4th. Phosphatic." All of these acidulous conditions are discernable by microscopic examination of the blood, demonstrating the several primary elements by the varied character of the parent gland cells.

It is not unreasonable to suppose that these various changes are the results of imperfect digestion and assimilation, varying in character according to the nature of the dietary, or functional powers of the patients—improvement in both of which may, in very many cases, be secured by the subject of this paper.

The nervous system, in both its centres and branches being also dependent for its integrity and healthful energy upon its proper nutrition, there is equally good reason for the opinion, that many of its derangements, such as hysteria, neuralgia, and even paralysis and meningitis, may be due to imperfect or deranged nutrition, as certainly as is delirium tremens, and in many such cases, the use of an agent for the purification of the nutrient fluid may be found directly influential in restoring a sound state of that organization, and likewise as in almost all other functional disorders, it may be found to be an important adjuvant to other essential remedies.

A case of unexpected relief to the mental functions, derived from its administration for a physical disease, will presently be reported.

In that peculiar pathological condition of the blood and cutaneous organization which is manifested by the production of numerous furuncles, commonly known as boils, the administration of sodæ sulphis, in combination with the carminative tonics, has proved, under my observation, a very perfect and rapid remedy. The same remark is applicable to another cutaneous disorder, dependant wholly upon gastric derangement. I refer to *urticaria.*

During the preparation of this essay, the most extensive and violent case of this disease that ever fell under my observation, came under my care. It was a lady aged 17, who had suffered greatly for several days with nausea, sleeplessness, an eruption covering almost the entire cutaneous surface, and accompanied with excessively violent itching. In twenty-four hours, a few doses of forty grains each, of sodæ sulphis, combined with carminative tonics, and a local external application of a solution of the salt, relieved all the symptoms to a great extent, and in forty-eight hours, they all wholly disappeared, leaving the patient in good health.

Another application of this salt, which I have found both highly interesting and valuable, is, in the case of infants, by whom their natural food, the mother's breast milk, is frequently rejected.

A dose of two to five grains, in combination with a few drops of the tinct. card. c., sweetened with a little syr. aurantii, has in many instances proved directly successful in causing a retention and assimilation of the stomach's contents, when administered soon after imbibition, thus greatly promoting the health and strength of the juvenile.

There are three forms of this salt, *viz.*, the sulphite, the hyposulphite, and the bi-sulphite—the first of which has been my principal dependence, though the others, when employed in proportionate quantities for the supply of the acid constituent, are equally useful. The only objection to the bi-sulphite is its being somewhat uncertain as to the proportions of acid contained in it, unless kept in solution, as a portion of the gas is liable to escape when exposed to the air in the crystaline form. —*Phila. Medical and Surgical Reporter.*

